# Annual Meeting of Constituents

**Published:** 1915-12-25

**Authors:** 


### METROPOLITAN HOSPITAL SUNDAY FUND.

### Annual Meeting of Constituents.

THE annual meeting of constituents of this Fund was held at the Mansion House on Monday, 20th inst., the Right Hon. the Lord Mayor (Sir Charles Cheers Wakefield), President and Treasurer, in the chair. Among those present were Sir C. Ernest Tritton, Sir William Church, K.C.B., Sir Henry Burdett, K.C.B., the Very Rev. Mgr. Carton de Wiart, Rev. Prebendary Ingram, Rev. J. G. Curry, Rev. H. A. Hall, Rev. A. A. Harland, Mr. Herbert Brooks, and Mr. A. F. Charrington.

The SECRETARY announced that letters of regret for absence had been received from the Archbishop of Westminster, Rev. J. W. Ewing, Lord Leith, Sir Savile Crossley, Surgeon-General Sir George Makins, F.R.C.S., and Sir R. L. Hamilton.

The minutes of the last meeting having been read and approved, the CHAIRMAN read the regulations controlling the annual meetings of the Fund.

A NOTABLE INCREASE IN THE COLLECTIONS.

Sir C. ERNEST TRITTON, Bart. (Vice-Chairman), proposed "That the Report of the Council for the year 1915 be, and is hereby, received and adopted." He said the Report was one which, in his opinion, could not fail to give satisfaction to all who were interested in the hospitals of London and in the work of the Hospital Sunday Fund. There were one or two features of particular interest in the Report which he would touch upon briefly. The first was the fact that our gracious King subscribed £100 to the Fund just before the collections were taken up on Hospital Sunday last. It was the first gift which the Fund had received from His Majesty, and it came most opportunely and, he was certain, did much towards increasing the gifts of his many loyal citizens, who were only too anxious to follow the good example which was set by the King on that occasion. The second point to which he wished to direct attention was that, in spite of the unprecedented claims from every quarter during the past year, the total amount for the Fund to distribute among the hospitals showed an increase of no less than £12,629 on that for the previous year. The Report now presented must be of great interest to those who perused it, because it clearly showed how the increase was made up. It was made up of collections, from church and chapel alike, larger than on previous occasions; it was contributed to by the rich and the poor, from the North, South, East and West of London. With very few exceptions, almost everywhere one saw an increase, either large or small, and all who were interested must feel most thankful to the clergy and ministers for the manner in which they had advocated the cause of the Fund, and to the congregations for the generous way in which they had responded to the appeal. Another point worthy of notice, as showing that every effort had been made to work the Fund on business principles, was that the percentage of expenses had now been reduced from 3.9 last year to 3.4, an indication of the efficient and economical service of Mr. Arnold James, the Secretary, and his assistant, Mr. Lander. The Fund was greatly indebted to those gentlemen. Lastly, he thought it must be a feature of this Report to see from the result what a deep hold the hospitals of London had upon the hearts of the inhabitants of the Metropolis. They realised, as the years passed, the great need there was for these institutions, which were doing such splendid work for the sick poor of our great city, and of the great need at this time for throwing open their wards, in the way they were now doing, to take in so many gallant wounded sailors and soldiers who had been upholding the honour of their country. They rejoiced to think that these hospitals were appreciated more than ever by the people of London, and that the Hospital Sunday Fund, which sought to do all it could to augment the incomes of those hospitals, still continued to hold a very warm place in the affections of our people. He concluded by paying a warm tribute to the Press of the Metropolis for the way in which they advocated the claims of the Fund.

Rev. G. MARTIN seconded the resolution with great pleasure. Though we were now living in a time of war, we were thankful for peace at home. It was a source of great satisfaction to all that, in spite of the many calls on the nation and the many claims on the congregations of churches and chapels, so great was the enthusiasm in support of the hospitals among both poor and rich, that there had been the satisfactory increase to which attention had already been directed. He had felt that in the past the Fund had not always received the support which it deserved, and he hoped that the new spirit of awakening evident in the nation would be continued, and would rise to even higher altitudes.

The resolution was carried.

Rev. W. H. HARDY HARWOOD proposed the following resolution: "That the Council for 1915 (including the Bishop of St. Albans, his Eminence Cardinal Bourne, Very Rev. the Chief Rabbi, Rev. Prebendary Cronshaw, M.A., Rev. Canon Allen Bell, M.A., Rev. John Clifford, D.D., Rev. J. G. Curry, B.A., Rev. F. K. Freeston, Rev. E. S. Hilliard, M.A., Rev. Prebendary Swayne, M.A., the Duke of Norfolk, K.G., Rt. Hon. Viscount Knutsford, Rt. Hon. Lord Leith of Fyvie, Sir Joseph Savory, Bt., Sir David Burnett, Bt., J. Astley Bloxam, Esq., F.R.C.S., J. R. Cooper, Esq., John H. Morgan, Esq., T. F. Rider, Esq., who retire under Law VIII.) be and is hereby re-elected for 1916, with the addition of the Rev. H. Newell Bate, M.A., the Rev. W. Perowne Holmes, M.A., the Rev. J. H. Wesley Kane, M.A., the Rev. F. Whitfield Daukes, M.A., the Rev. Richard Peek, M.A., Edward Dent, Esq., and Edward Eyre, Esq., to fill vacancies." There were, he said, ten vacancies, but five of these were clerical and five lay. More clerical than lay nominations were received, and the gentlemen were chosen who had shown themselves keenly interested in the Fund, and would be as great helpers inside the Council as they had so far been outside. He wished beforehand to congratulate those who would now become members of the Council, for after twenty-five years' experience in the operative part of the Fund he wished to testify to the absolute fairness which he had always found in the activities of the Council of the Fund. There was the strong desire to serve with one heart and mind the great hospitals of London, and he welcomed the new colleagues who had been elected to the Council.

Mr. H. E. GOLDING (St. Matthias', Earl's Court) seconded, and it was carried unanimously.

Sir HENRY BURDETT, K.C.B., proposed, with great pleasure, the following resolution: "That the 25th of

June be fixed for Hospital Sunday, 1916, and that the cordial co-operation of all ministers of religion within the Metropolitan area be again invited in the usual way." In doing so, he said his first duty was to express thanks and congratulation. In this year of war, at the beginning of January, few people had heart enough and belief enough in the voluntary system to think it would be possible that the results attained by this Fund, as testified to-day, could possibly have ensued. That the congregational collections in such a year, and the number of churches contributing, should both show an increase was a remarkable fact. It was a fact which those who read the events of the times and took an interest in the development of the nation must recognise as markedly testifying to what all who moved about knew, that there never was a time, probably, in the history of this nation, when everybody everywhere, and of every class, wanted to do something, as a mere matter of filling their part in this great war, which had underlying it such important issues for the men and women of the nations. This was the forty-third year of the Hospital Sunday Fund in London, and it showed 2,186 churches and congregations collecting for the Fund—the greatest number in its history. Further than that, the actual amount was £39,547, and the increase amounted to £10,847. Surely those figures and facts were the best thanks he could offer on behalf of the Council, and also on the part of the people of London, to the clergy and other ministers of religion for the efforts they must have put forward, and for the willing supporters from whom they had collected, many, no doubt, for the first time. It was also interesting in this connection to remember that Hospital Sunday had languished for years, not only in London, but throughout the country. In fact, it was becoming almost a commonplace for people to say it had "had its day." This year Hospital Sunday had shown new life almost everywhere. That was further testimony to the feeling of the nation and the spirit in which our people were working and living to-day. That spirit had had another wonderful effect. These Funds were great exemplars; they were leaders for the givers in the nation. It was a fact that this organisation, and also that of the King's Fund, had extended the area of givers enormously, so much so that the people had been successfully led to acquire the habit of giving to hospitals for the sick. And whatever else most people now did when they had money to give away, they first put hospitals down on their list. Again, the most wonderful collections for the Red Cross, he claimed—and the evidence was quite definite—meant that the education and teaching through the Churches, through the great Funds, and through leaders trusted by the public had prepared the way for these gifts to the Red Cross. He hoped the clergy and ministers of religion generally would feel encouraged by the results of 1915. He asked them to put their shoulders at the wheel in 1916, to advance the business of Hospital Sunday, and to resolve to maintain the collections at not less than the amount realised this year.

Sir WM. CHURCH, Bt., K.C.B., seconded, and the resolution was unanimously accepted.

Sir MARCUS SAMUEL proposed in cordial terms a hearty vote of thanks to the Lord Mayor for his help to the Fund. This was carried by acclamation, briefly acknowledged, and the meeting terminated.

